# Is the value of a life or life-year saved context specific? Further evidence from a discrete choice experiment

**DOI:** 10.1186/1478-7547-6-8

**Published:** 2008-05-20

**Authors:** Duncan Mortimer, Leonie Segal

**Affiliations:** 1 Centre for Health Economics, Faculty of Business & Economics, Monash University, Melbourne, Australia; 2 Faculty of Nursing & Midwifery, University of South Australia, Adelaide, Australia

## Abstract

**Background:**

A number of recent findings imply that the value of a life saved, life-year (LY) saved or quality-adjusted life year (QALY) saved varies depending on the characteristics of the life, LY or QALY under consideration. Despite these findings, budget allocations continue to be made as if all healthy life-years are equivalent. This continued focus on simple health maximisation is partly attributable to gaps in the available evidence. The present study attempts to close some of these gaps.

**Methods:**

Discrete choice experiment to estimate the marginal rate of substitution between cost, effectiveness and various non-health arguments. Odds of selecting profile B over profile A estimated via binary logistic regression. Marginal rates of substitution between attributes (including cost) then derived from estimated regression coefficients.

**Results:**

Respondents were more likely to select less costly, more effective interventions with a strong evidence base where the beneficiary did not contribute to their illness. Results also suggest that respondents preferred prevention over cure. Interventions for young children were most preferred, followed by interventions for young adults, then interventions for working age adults and with interventions targeted at the elderly given lowest priority.

**Conclusion:**

Results confirm that a trade-off exists between cost, effectiveness and non-health arguments when respondents prioritise health programs. That said, it is true that respondents were more likely to select less costly, more effective interventions – confirming that it is an adjustment to, rather than an outright rejection of, simple health maximisation that is required.

## Introduction

A number of recent findings imply that the value of a life saved, life-year (LY) saved or quality-adjusted life year (QALY) saved varies depending on an increasingly diverse set of non-health contextual factors that includes characteristics of the patient and intervention [1]. For example, a number of studies suggest that the value of outcomes varies according to the age or life-stage of recipients [2-5]. These age-based distributive preferences might arise from one of several motivations including capacity to benefit [6-8], interaction between capacity to benefit and net productive contribution to society at different life-stages [9], deviations from a 'fair innings' [10], or 'vicarious utility' associated with an emotive response to saving particular *types *of people such as children or their parents [11].

The significance of such findings is two-fold. First, variation in the non-health characteristics of outcomes might explain some of the substantial variation in published estimates for the value of a life saved, LY saved or QALY saved. Estimates of willingness to pay for reductions in risk of death expressed in 1998 AUD equivalents range from AUD1.8 to AUD4.2million [12] but the range of values becomes even wider when estimates based on willingness to accept for an *increased *risk of death and compensating wage differentials are taken into consideration [13]. If some of this variation in such estimates can be attributed to systematic variation in health or non-health arguments in the objective function (rather than to elicitation biases, error or framing effects), then this might increase confidence in the use of monetary values for priority setting [14]. Second, if the value of a life, LY or QALY is context specific, then efficient allocation of resources demands a departure from simple health maximisation and the assumption of 'distributive neutrality' [5]. Note, for example, that – in pursuit of efficiency gains – we might fund interventions for children at a less stringent threshold (eg, higher cost per QALY) than interventions for the elderly if health gains for children can be shown to be more highly valued than health gains for the elderly. Previous attempts to estimate the dollar-value of a QALY have focused on the tradeoffs between cost, and health attributes including duration, various dimensions of health-related quality of life and severity [15-18], leaving value-weights reflecting the tradeoff between health and non-health attributes "to be super-imposed by the decision maker" [[17] p1050].

To date, attempts to value-weight funding thresholds or outcomes [19] have typically adjusted for only a narrow subset of potentially relevant non-health characteristics such as distribution [20], age [9] or severity [21]. Mortimer [22] suggests that this is partly attributable to the complexity of simultaneously adjusting for even a relatively narrow set of non-health characteristics and partly due to data gaps with respect to the tradeoffs between potentially relevant non-health characteristics (as opposed to the trade-off between either cost or effectiveness and one or other of these potentially relevant non-health arguments). In an attempt to address these gaps, we conduct a discrete choice experiment to estimate the marginal rate of substitution between cost, effectiveness and various non-health arguments including the life-stage of beneficiaries, the extent to which beneficiaries have contributed to their illness via voluntary adoption of risky lifestyle, the extent to which beneficiaries will contribute to the cost of the intervention, the type of intervention (lifestyle versus medical), and the aim of the intervention (cure versus prevention).

## Methods

### Experimental design

Potentially relevant attributes were identified from a review of the literature [eg. [1-11]; [15-22]], yielding a set of more than fifty potentially relevant characteristics of interventions including incremental cost; budget impact; out-of-pocket costs; total cost [23]; the magnitude and timing of mortality gains; the magnitude, duration and timing of quality of life gains; the magnitude, duration and timing of non-health benefits including productivity gains [24]; and an almost innumerable number of patient characteristics including severity [25]; prognosis; age or life-stage; fault; marital status; contribution to society; race; sexuality; gender; responsibility for others; wealth; lifestyle; whether or not the patient has a criminal record; and parental status [26]. The study team considered using labels (for interventions or for the condition or problem being targeted) as a 'short-hand' that might capture variation over multiple attributes but this option was rejected in favour of unlabelled alternatives in which each level on each attribute of interest was explicitly described. This strategy was chosen to minimise labelling effects that might limit the extent to which findings could be generalised to different interventions targeting different conditions/problems [27] and to permit estimation of the independent effect of each attribute of interest.

Due to the sheer number of potentially relevant attributes, the study team decided to narrow the scope of the experiment to focus on eliciting preferences over *life-saving *interventions differentiated by a subset of patient and program characteristics. The attributes and levels included in our discrete choice experiment therefore provide only a partial description of each program but are intended to provide a complete description of differences between alternative programs. The validity of parameter estimates on each of the included attributes is therefore dependent on the assumption that respondents evaluated competing programs as equivalent with respect to excluded attributes and that the effect of each excluded attribute is orthogonal to the effect of each included attribute. Put another way, the derivation of a universal set of value-weights was not considered practical given the sheer number of potentially relevant attributes and we instead consider tradeoffs between health and non-health attributes for programs that are equivalent with respect to the majority of patient characteristics including severity, sexuality and prognosis, and with respect to many program characteristics including quality of life; the timing of costs and consequences; and the magnitude, timing and duration of non-health benefits.

Several versions of the questionnaire were piloted in a small convenience sample of tertiary educated but otherwise diverse individuals to identify potential problems with comprehension and interpretation and to reduce the set of attributes to a size consistent with the information processing capacity of respondents. "Because of the problem of cognitive overload, there is always a trade-off between comprehensiveness and realism on the one hand and the ability of subjects to comprehend and evaluate" on the other [[28] p152]. When the number of information 'elements' is too large, individuals have a tendency to focus upon only one element or attribute and may become inconsistent in their appraisal of competing programs. While data regarding the trade-off between task complexity and realism in the context of choice experiments are lacking [29], Froberg and Kane [30] suggest that the choice set should be defined over no more than nine attributes because research [31] "has shown that humans can process simultaneously only five to nine pieces of information" [[30] p. 346]. Note also that very few choice experiments to value health care programs have included more than eight attributes [32]. The pilot surveys varied the attributes, levels, choice format (discrete choice versus a graded pairs format [15] with respondents asked to rate the intensity of their preference for their preferred alternative) and wording of a limited number of scenarios, with respondents encouraged to talk through their decision-process and to provide a rationale for each decision.

Table 1 lists the final set of attributes and levels for the health survey. The final set of attributes excluded a number of attributes considered in the pilot surveys including the presence and severity of side-effects associated with an intervention, whether the intervention is in current use or a new technology, whether the person providing the intervention is an allied health professional or a medical doctor, and the level of effort that would be required of the patient to comply with the prescribed treatment regimen. Attributes were excluded if nested within other attributes or if they were largely ignored or deemed irrelevant by respondents in the pilot surveys (eg. level of effort to comply, whether or not the intervention is in current use). Levels for each attribute were initially selected to be plausible and actionable in the opinion of the study team but were modified in response to feedback from the pilot surveys and to keep the size of the choice set to a manageable level. While it is recognised that the number of levels for each attribute falls short of capturing the full range of variation in real-world programs, the much larger sample size that would have been required to estimate main effects for a model with four or more levels on each of eight attributes was not feasible. The final set of attributes and levels defines a universe of 4096 profiles (2*2*2*4*2*4*4*4). The Orthoplan procedure of SPSS was used to generate the bare minimum of 32 profiles over which preferences were elicited in order to estimate main effects.

**Table 1:** Attributes and levels for health programs

**Attributes**			**Levels**	
**Number**	**Meaning**	**Label**	**Code**	**Label**

1	Does individual behaviour cause the problem requiring the intervention?	Fault	0	No
			1	Partly
2	What is the purpose of the intervention?	Cure	0	Prevention
			1	Treatment
3	What type of intervention is it?	Medical	0	Lifestyle
			1	Medical
4	According to the evidence: How many lives will it save per year?	Lives	0	10
			1	20
			2	30
			3	40
5	How good is this evidence?	Evidence	0	Limited
			1	Strong
6	How much will it cost?	Cost	0	$500,000
			1	$1,000,000
			2	$5,000,000
			3	$10,000,000
7	How much will patients have to contribute?	Private	0	Nothing
			1	Quarter of the cost
			2	Half the cost
			3	All of the cost
8	At what life-stage are those who stand to benefit from the program?	AgeGrp	0	Young children
			1	Young adult
			2	Working-age adult
			3	Older-age retiree

Discrete choice scenarios were constructed as a two-alternative forced choice to obtain 32 scenarios that were then randomly distributed across four versions of the health questionnaire. An example of the discrete choice scenarios presented to respondents is given in Table 2. Each version of the questionnaire included eight health scenarios plus one hold-out pair with a dominant profile to provide a check that respondents understood the task and were making rational choices. The questionnaire included instructions to 'notice the bolded differences between the two programs, indicate which program you would prefer the government to implement and briefly comment on your reasons'. The option for respondents to briefly explain their choice for each scenario was provided as a further check on rationality. Respondents also received a separate sheet with a list of examples to assist with interpreting terms that were identified by respondents to the pilot surveys as being too abstract to provide a basis for choices between programs without further explanation. The questionnaire included a cross-sector survey alongside the health survey, also with eight scenarios plus one hold-out pair but requiring comparisons across health, transport, environment and workplace programs. Methods and results for the cross-sector survey are described elsewhere [33].

**Table 2:** Example scenario from the health survey

**Q3. Would you prefer the government to implement 3A or 3B? **(Pair 29)		
	**KEY FEATURES**	
	↓	
3A	A **medical **program to prevent a health problem from occurring in **working-age adults**.	
	The problem is **not **caused by patients' behaviour.	
	Based on strong evidence, the program is expected to save **40 **lives every year.	
	It will cost **ten **million dollars.	
	Patients will pay half of the cost of their participation.	

3B	A **lifestyle **program to prevent a health problem from occurring in **young adults**.	
	The problem is **partly **caused by patients' behaviour.	
	Based on strong evidence, the program is expected to save **20 **lives every year.	
	It will cost **one **million dollars.	
	Patients will pay half of the cost of their participation.	
Tick ONE box to indicate which program you prefer:		
	**∋**	**∋**
	3A	3B
Briefly, what are your reasons for this decision?		
......................................................................................................................................................		

### Survey

The survey was distributed via Australia Post to 4,000 addressees randomly selected from the Australian WhitePages telephone directory. Four versions of the questionnaire were distributed, with each of the 4,000 addressees randomly assigned to receive one of the four versions. A total of 274 respondents provided a response to at least one question and returned the instrument. An additional 176 questionnaires were returned unopened and marked either 'return to sender' or 'incorrect address' and a further 21 addressees excluded themselves due to age/health (n = 4), because they found the questionnaire difficult to understand (n = 6), because they were too busy to participate (n = 1), because they were deceased (n = 1) or for unspecified reasons (n = 9). Of the 274 respondents, 37 respondents failed to provide a response on at least one choice scenario in the health survey (90 missing values on the dependent variable); three of which failed to provide a response on any of the choice scenarios in the health survey (accounting for 21 of the 90 missing values on the dependent variable). After deletion of 90 missing values on the dependent variable, 2,376 stated preferences over alternative health programs from 271 respondents were available for analysis.

Respondents to the questionnaire were from localities (post office areas) with a significantly higher SEIFA (Socio-Economic Indices for Areas) index of socio-economic disadvantage when compared to 2001 Census of Population and Housing data (t = 3.285, p = 0.001). This would suggest that the sample over-represents persons resident in areas with relatively few low income families working in unskilled occupations (ABS, 2003). Similar differences were observed for the SEIFA index of economic resources (t = 7.237, p < 0.000) and the SEIFA index of education and occupation (t = 6.463, p < 0.000). Comparisons with census data also suggested that the survey sample over-represented persons aged 50 years or over and individuals with preferential access to health care under either private insurance coverage or a government health care card for eligible residents on a low income, parenting/carer allowances or unemployment benefits. Table 3 describes and compares characteristics of the Australian population and of the 274 survey respondents. Table 3 also reports the number of respondents who failed to complete one or more of the questions relating to individual and small-area characteristics (eg. six respondents failed to report their gender and nine respondents failed to report a postcode for the purposes of matching residential location against small-area characteristics). Missing values on individual and small-area characteristics were imputed using best-subsets regression on age, gender, parent/not, birthplace and/or health care card status.

**Table 3:** Characteristics of Australian population versus survey sample

Version	Population: (%)	Survey: N(%)
Version A	-	65 (23.7)
Version B	-	61 (22.3)
Version C	-	83 (30.3)
Version D	-	65 (23.7)
Gender		
Male	(48.9)^†^	126 (46.0)
Female	(51.1)^†^	142 (51.8)
Missing	-	6 (2.2)
Age Group		
15–19 yrs	(8.9)^†^	0 (0.0)
20–29 yrs	(17.2)^†^	15 (5.5)
30–39 yrs	(19.1)^†^	35 (12.8)
40–49 yrs	(18.6)^†^	48 (17.5)
50–59 yrs	(14.9)^†^	62 (22.6)
60–69 yrs	(9.8)^†^	41 (15.0)
70–79 yrs	(7.6)^†^	49 (17.9)
80+yrs	(3.9)^†^	18 (6.6)
Missing	-	6 (2.2)
Birthplace		
Australia	(76.6)^†^	205 (74.8)
Other	(23.1)^†^	61 (22.3)
Missing	-	8 (2.9)
Health Care Card		
Yes	(30.0)^‡^	107 (39.1)
No	(70.0)^‡^	158 (57.7)
Not Sure	-	1 (0.4)
Missing	-	8 (2.9)
Parent		
Yes	-	222 (81.0)
No	-	45 (16.4)
Not Sure	-	1 (0.4)
Missing	-	6 (2.2)
SEIFA Index of Socio-Economic Disadvantage		
> 962 (Quartile_1_)	(75.0)^	210 (76.6)
> 1000 (Quartile_2_)	(50.0)^	147 (53.6)
> 1044 (Quartile_3_)	(25.0)^	88 (32.1)
Missing	-	9 (3.3)
SEIFA Index of Economic Resources		
> 910 (Quartile_1_)	(75.0)^	230 (83.9)
> 954 (Quartile_2_)	(50.0)^	191 (69.7)
> 1023 (Quartile_3_)	(25.0)^	109 (39.8)
Missing	-	9 (3.3)
SEIFA Index of Education and Occupation		
> 925 (Quartile_1_)	(75.0)^	237 (86.5)
> 959 (Quartile_2_)	(50.0)^	181 (66.1)
> 1017 (Quartile_3_)	(25.0)^	118 (43.1)
Missing	-	9 (3.3)

^†^**Source**: ABS Census of Population and Housing 2001, Basic Community Profile (Catalogue No. 2001.0), Commonwealth of Australia, 2002 [53].

^‡^**Source**: ABS National Health Survey 2004–05: Summary of Results (Catalogue No. 4364.0), Commonwealth of Australia, 2006 [54].

^**Source**: ABS Census of Population and Housing 2001, Socio-Economic Indexes for Areas (Catalogue No. 2039.0), Commonwealth of Australia, 2003 [55].

A higher number of C-version questionnaires were returned than A-, B- or D-version questionnaires, though there was no significant association between assignment to questionnaire version in those sent the questionnaire and response (χ^2 ^= 5.663, df = 3, p = 0.129). There was also no significant association between assignment to questionnaire version in those returning the questionnaire and proportion aged over 50 (χ^2 ^= 1.855, df = 3, p = 0.603), gender (χ^2 ^= 2.403, df = 3, p = 0.493), health care card status (χ^2 ^= 4.026, df = 3, p = 0.259), country of birth (χ^2 ^= 1.098, df = 3, p = 0.777), SEIFA index of socio-economic disadvantage (F = 2.013, df = (3,261), p = 0.112), SEIFA index of economic resources (F = 2.324, df = (3,261), p = 0.075), SEIFA index of education and occupation (F = 1.122, df = (3,261), p = 0.341) or whether the respondent reported having children (χ^2 ^= 3.016, df = 3, p = 0.389). To ensure that the higher relative frequency of C-version responses do not exert undue influence on parameter estimates, probability weights (pweights) were applied to each choice scenario with the pweight for each choice scenario derived as the inverse of the relative frequency of response for that choice scenario.

A small number of respondents (varying in age from 31 to 88 years and predominantly born in Australia) selected the dominated profile from the hold-out pair in the health survey (8/274). The hold-out pair was included with the intention of providing a test of whether stated preferences could be considered rational. However, the reasons given by respondents for selecting a dominated profile suggested that these respondents are more appropriately characterised as careless than irrational. For example, one respondent (ID: 2) selected a dominated (more expensive) profile but stated his/her reason for selecting this profile as "costs less". This respondent provided a response and an explanation of his/her reasoning for all but one scenario and refused to make a choice for the remaining scenario because "young children and young adults are equally important" and he/she "could not make a decision". Likewise, another respondent (ID: 102) selected a dominated (less effective) profile but stated her reason for selecting this profile as "saves more lives for equal cost to government, based on strong evidence". The majority of respondents who selected dominated profiles provided detailed explanations of their reasoning that could not be considered irrational.

It is worth emphasising that "censoring is unnecessary and perhaps detrimental" [[34] p160] for random errors whereas the inclusion of non-random errors will tend to bias results [35]. While non-random errors that reflect "preference structures that are not compatible with (random) utility theory or a failure to comprehend how to use the rating tool" [[34] p160] may be present in our dataset, it does not appear that the errors described above fall into this category. Rather, the errors described above are more appropriately characterised as 'lapses of attention' that are unlikely to bias results. For this reason (and because only a very small number of respondents selected dominated profiles), the study team decided not to censor data from respondents who selected a dominated profile.

More generally, reasons for selecting one profile over another for each choice scenario were classified and paired with illustrative statements in a subsample of over 100 respondents. This subsample of respondents was presented with 954 opportunities to provide a rationale specifically relating to a choice scenario. Each respondent was also given the opportunity to make general comments relating to the questionnaire and/or their responses. The attributes/levels included in the discrete choice experiment provided a framework for interpretation and coding of rationales. Table 4 provides a classification of rationales and reports a simple count of the number of times each rationale was mentioned in the subsample, together with one or more examples transcribed from questionnaires. The explanations given in support of stated-preferences suggested that respondents were making principled decisions based on due consideration of the alternatives presented to them.

**Table 4:** Classification of reasons given for stated-preferences

Reason	Count	Examples
More effective/outcomes better	152	"Greater number of lives saved" (ID:75).

More cost-effective	148	"Same number of lives expected to be saved at half the cost" (ID: 86).
		"Low cost per expected benefits mitigates low evidence" (ID: 5).
		"Better value for money" (ID: 17).
		"Greater impact for dollars invested" (ID: 21).
		"It makes sense to save more lives for the same cost" (ID: 73).

Prevention better than cure/treatment	108	"Prevention is better than cure" (ID: 24).
		"Prevention is better than cure especially in young" (ID: 64).
		"Prevention is better than cure – is initially maybe more costly but in the long term will be effective and economical because less people will need treatment" (ID: 70).
		"Better to stop something happening than to clean up the mess later" (ID: 72).
		"May be limited evidence, but prevention is better than treatment" (ID: 76).

High quality evidence	145	"Strong evidence – therefore more likely to succeed" (ID: 16).
		"Strong evidence vs limited evidence" (ID: 89).
		"Strong evidence that it will work" (ID: 90)

Lifestyle better than medical	45	"Lifestyle may give a better outcome over time" (ID: 1).
		"I always prefer lifestyle to medical. It is more effective and cheaper in the long term" (ID: 24)
		"Most illnesses are caused by lifestyle factors. Only lifestyle changes can reverse them. Medicine causes many problems we see today or at least contributes" (ID: 52).

Medical program better than lifestyle	24	"A medical program seems more likely to be followed through because the onus is less on the patient" (ID: 67)
		"I would favour a lifestyle program in preference to medical, if results the same" (ID: 101).
		"Medical is essential – lifestyle is self inflicted" (ID: 29).

Young children a priority	140	"Young children grow into young adults and problems are easier to fix in young children" (ID: 60)
		"Young children deserve the right to have the best treatment available" (ID: 34).
		"Elderly have had their life and children have it all in front of them – they are the Australia of tomorrow" (ID: 29)
		"We should spend more on keeping young people healthy rather than keeping elderly people alive" (ID: 71).
		"Helping children is very important especially if it's fully funded so children aren't prevented from participation because of socio-economic factors" (ID: 82).

Young adults a priority	52	"Young adults grow into elderly adults so it would be better to treat young adults who would save the govt money and be more useful in the workforce till they age" (ID: 60).
		"We have to invest in the young adults as they are our future, even at a higher cost. The elderly have lived some of their lives already" (ID: 96).
		"Prefer young adults be treated before elderly so their lives may be extended for the community benefit" (ID: 19)

Working age adults a priority	33	"Working adults may be able to stay in work force for a longer period" (ID: 74).
		"Working age adults likely to be responsible for young children" (ID: 87).
		"Working age adults have a lot of responsibility – often the sole bread winners; supporting them is better for our society" (ID: 2).
		"The working age people are required to provide for others and need to be healthy" (ID: 40).
		"Working adults are tax payers" (ID: 47).

Elderly a priority	22	"The elderly need help now. By the time the working age adults develop their problem, a cure may have been found" (ID: 67).
		"Most elderly worked and paid taxes most of their working lives" (ID: 101).
		"Elderly usually have longstanding health problems anyway, less inclined to change lifestyle" (ID: 13).
		"I know older people suffer more than they should. GP's don't care about chronic pain. Help elderly people, who are usually on very limited incomes, more" (ID: 4).
		"To assist the elderly and hopefully provide an improved quality of life" (ID: 16).

Not at fault should be given priority	53	"Prefer to help when problem is not caused by patient's behaviour" (ID: 35).
		"If the problem is partly caused by patients' behaviour, then they should pay for the program" (ID: 48)
		"Caused by their behaviour makes something very low priority" (ID: 84).

Higher patient contribution	54	"If people pay nothing they will not change the ways that cause their problem. Ownership is essential" (ID: 52)
		"People must be responsible for some help costs – Medicare is out of control!" (ID: 10).
		"If the patient is partly responsible they should partly pay for the treatment" (ID: 40).
		"People don't appreciate or necessarily stick to the things they get for free" (ID: 18).

Lower or no cost to patient/participant	35	"No cost to participants. To expect young adult to pay for a lifestyle program may prohibit some from being able to participate" (ID: 86).
		"Available to all as it's free" (ID: 18).
		"Government should be prepared to arrange and fund public health initiatives" (ID: 103).

Lower cost to government/tax payers	8	"Lower cost to government" (ID: 51).
		"No cost to tax payers" (ID: 49).

Lower cost/cheaper	41	"Cheapest to implement" (ID: 96).

### Data analysis

The survey described above was designed with the primary aim of relating preferences over profiles to variation across profile attributes. However, in order to obtain observations over a sufficient number of profiles, respondents were *randomly *allocated to one of four versions of the instrument such that different respondents were faced with different choice scenarios. For the choice between two profiles, the dependent variable is binary and a single logit function describes the odds of selecting profile A relative to profile B. The general model is then defined as

L(**C**_ij_) = g (β**x**_ij_, δ**p**_ij_, γ**z**_i_) + ε_ij_

ε_ij _= v_i _+ u_ij_

Where L(**C**_ij_) = ln Pr(**C**_ij_)/(1- Pr(**C**_ij_)) such that L(**C**_ij_) gives the log-odds ratio corresponding to the probability that individual i selects profile B given the value of **x**, **p **and **z **for profile B as compared to profile A. **x **is a vector of difference scores designating each level of each attribute for profile B as compared to profile A in scenario j. **p **is the price difference for profile B as compared to profile A in scenario j. **z **is a vector of individual characteristics (such as age, insurance status and whether the individual has any children) interacted with a scenario-specific effect to distinguish **z **variables from respondent-specific effects. ε_ij _is a composed error term comprising: within-individual errors (v_i_) arising from uncontrolled heterogeneity in perceived profile attributes and purely stochastic elements, and between-individual errors (u_ij_) reflecting uncontrolled heterogeneity in individual characteristics, uncontrolled heterogeneity in perceived profile attributes and purely stochastic elements.

The simplest approach to estimation is to assume that the composed residuals are iid and to estimate a population-average logistic regression model. In the present study, however, observations are clustered by respondent such that residuals might be independent between clusters but may not be independent within clusters. The robust Huber/White sandwich estimator is frequently used to adjust for clustering in situations where the intra-cluster correlation coefficient is significantly greater than zero. While this approach delivers robust standard errors suitable for calculating confidence intervals, it does not render an inconsistent model (due to failure to control for respondent-specific effects) consistent [36]. The random effects error components model explicitly accounts for cluster-specific effects and provides a variance partition coefficient: *σ*_*v*_^2^/(*σ*_*v*_^2 ^+ *σ*_*u*_^2^), to quantify the proportion of residual variance attributable to respondent-specific effects [37]. For the present study, the choice between the random effects model and the population-average model will be treated as an empirical question based on the significance of respondent-specific effects.

Before conducting the analysis described above, the levels of categorical attributes were dummy coded and then expressed as a difference between profile B and profile A. Incremental cost of profile B as compared to profile A and the private contribution to this incremental cost were expressed as a difference score in current AUD at the time of data collection. At the commencement of data collection for the present study in July 2005, conversion rates to selected major currencies were 0.63 Euros per AUD, 0.42 United Kingdom Pounds per AUD and 0.75 US Dollars per AUD. Incremental effectiveness of profile B as compared to profile A was expressed as a difference score in terms of lives saved. Incremental effectiveness was also expressed in terms of LYs saved in an attempt to control for duration and to permit willingness to pay to be calculated for LYs as well as lives. An estimate of LYs saved was obtained by combining estimates of population by age and sex [38] with life-expectancies at each life-stage for the Australian population [39]. This calculation required an exact age to be specified for each life-stage as follows: 'young children': 5 yrs, 'young adults': 18 yrs, 'working-age adults': 40 yrs, 'older-age retirees': 70 yrs.

### Estimating WTP

One of the primary reasons for employing discrete choice methods in the present study is that willingness to pay (WTP) for a life and LY saved can be inferred from the trade-offs between attributes that respondents make when choosing one program over another. Under random utility theory (RUT), the utility difference between profile B and profile A is an unobserved latent variable that is closely related to response variable from our discrete choice experiment: **C**_ij_. The utility difference between profiles can then be approximated from the regression such that U_iB _- U_iA _= g (β**x**_ij_, δ**p**_ij_, γ**z**_i_) + ε_ij_.

The marginal effect of a change in the j^th ^profile therefore provides an estimate of the marginal utility derived from that change. For linear regression models, the marginal effect of a change in an attribute would be given by the estimated regression coefficient on that attribute. In the context of the logistic regression model, marginal effects vary with the value of the covariates such that MU_j _= ∂ U_B _- U_A_/∂ x_j _= g (X'β) * β_j _where g (.) refers to the logistic cumulative distribution function, x_j _is the attribute of interest and all other covariates are held at either their mean or median values or are specified so as to reflect a profile of particular interest. The willingess to trade between two profiles or attributes with utility held constant (along an indifference curve) is defined as the marginal rate of substitution and can be derived as the ratio of marginal utilities: MRS_2,1 _= - d x_2_/d x_1 _= (∂ U_B _- U_A_/∂ x_1_)/(∂ U_B _- U_A_/∂ x_2_) = MU_1_/MU_2_. In other words, the marginal rate of substitution or willingess to trade between preventative and curative interventions or between an intervention for young adults and an intervention for the elderly or between any two of the attribute levels included in the discrete choice experiment described above can be approximated as the ratio of the relevant marginal effects. Likewise, willingness to trade between price and the outcome of interest gives us an estimate of willingness to pay for the outcome of interest and can be derived by dividing the marginal effect associated with a change in incremental effectiveness by the marginal effect associated with a change in incremental cost. Phillips [40] and others have suggested that this approach is likely to deliver more realistic estimates than directly eliciting WTP values for outcomes or programs.

For the present study, WTP estimates can only be derived for a life or LY saved because the choice set was delimited to *life-saving *interventions with negligible quality of life effects. To calculate WTP for a LY gained, we first obtain the marginal effect corresponding to a one LY change in incremental effectiveness with other attribute levels held constant and divide this through by the marginal effect corresponding to a one dollar change in incremental cost. To calculate WTP for a program targeted at one age-group rather than another, we obtain the marginal effect corresponding to a movement between levels of the life-stage attribute and divide this through by the marginal effect corresponding to a one dollar change in incremental cost. In this way, WTP for different types of health program can be derived and the effect of non-health arguments or 'context' can be inferred from marginal effects calculated from estimated regression coefficients.

## Results

Binary logistic regression was undertaken to identify attributes from Table 1 and respondent or small-area characteristics from Table 3 that might explain stated preferences over profiles. The intra-cluster correlation coefficient for profile choice was not significantly greater than zero (ICC = 0.000, 95%CI: 0.00, 0.02) such that adjustment for clustering by individual is unnecessary in the present study. Results from the random effects error components model (not reported here) confirm that the variance partition coefficient: *σ*_*v*_^2^/(*σ*_*v*_^2 ^+ *σ*_*u*_^2^), is approximately zero, implying that the proportion of residual variance attributable to respondent-specific effects is also approximately zero [37]. Further adjustment for (non-existent) respondent-specific effects using either conditional fixed effects or random effects error components models is therefore unnecessary and results from the population-average model reported in Table 5 adequately characterise preferences over profiles.

**Table 5:** Parameter estimates for population-average model using robust regression with pweights

Predictor	β	SE	z	Sig.	β	SE	z	Sig.
	Lives saved				Life-years saved			

Medical(B – A)				ns				ns
Cure(B – A)	-0.8476	0.110	-7.68	0.000	-0.8330	0.105	-7.93	0.000
AgeGrp_(B – A)^†^			χ^2 ^= 130	0.000			χ^2 ^= 28.9	0.000
AgeGrp1(B – A)^†^	1.2894	0.148	8.72	0.000	0.7448	0.144	5.17	0.000
AgeGrp2(B – A)^†^	0.5936	0.138	4.30	0.000	0.3001	0.132	2.28	0.023
AgeGrp4(B – A)^†^	-0.3810	0.110	-3.45	0.001	0.0187	0.130	0.14	0.886
Evidence(B – A)	0.6857	0.093	7.34	0.000	0.6572	0.093	7.05	0.000
Fault(B – A)	-0.5822	0.097	-5.98	0.000	-0.6560	0.104	-6.31	0.000
$Private(B – A)^	-0.0055	0.002	-2.49	0.013	-0.0077	0.002	-3.59	0.000
Effect(B – A)^‡^	0.0338	0.004	8.43	0.000	0.0006	0.000	7.53	0.000
$Cost(B – A)^	-0.0060	0.001	-4.50	0.000	-0.0057	0.001	-4.22	0.000
HlthCard*Q	-0.0456	0.018	-2.52	0.012	-0.0454	0.018	-2.51	0.012
SIEFA_Econ*Q/1000	0.0693	0.022	3.15	0.002	0.0794	0.022	3.58	0.000
(Constant)	-0.3415	0.118	-2.89	0.004	-0.3995	0.117	-3.43	0.001

				N = 2329				N = 2329
				Wald χ^2 ^= 352.32, df = 11, p = 0.000				Wald χ^2 ^= 346.91, df = 11, p = 0.000
				Log-likelihood = -1234.69, Pseudo R^2 ^= 0.2350				Log-likelihood = -1239.33, Pseudo R^2 ^= 0.2321

^Dollar values expressed in AUD100,000s.

^†^Reference category is 'working-age adults'. First, second and fourth dummies denote 'young children', 'young adults' and 'older-age retirees', respectively. Joint significance of dummies evaluated using Wald statistic on chi-square distribution.

^‡^Effect(B – A) gives the incremental effectiveness of profile B compared to profile A defined in terms of terms of lives saved for the 'lives-saved' model and life-years saved for the 'life-years saved' model.

With regards to respondent and small-area characteristics, only health care card status (HlthCard) and the SEIFA Index of Economic Resources (SEIFA_Econ) reached individual significance. In contrast, the majority of profile attributes included in the experiment were individually or jointly significant – confirming their relevance in explaining preferences over health programs. That said, the Medical(B – A) attribute failed to reach individual significance in all models such that the medical/lifestyle distinction did not influence profile choice in our experiment. Coefficients on individual levels of multinomial attributes such as: AgeGrp4(B – A), also failed to reach individual significance in some models. Multinomial attributes coded as sets of dummy variables were retained or excluded on the basis of joint significance, with each level of a jointly significant set of dummies retained regardless of individual significance.

Table 5 reports parameter estimates for the population-average model with the incremental effectiveness of profile B as compared to profile A expressed in terms of lives saved and LYs saved. Interpretation of the parameter estimates is straightforward but it should be remembered that the estimated logit function describes the odds of selecting profile B relative to profile A. For the lives saved model, respondents were more likely to select less costly, more effective interventions with a strong evidence base where the beneficiary did not contribute to their illness. Results also suggest that respondents preferred prevention over cure. Interventions for young children were most preferred, followed by interventions for young adults, then interventions for working age adults and with interventions targeting the elderly given lowest priority. While these results and the implied marginal rates of substitution are consistent with expectations, results also suggest that – despite providing more output per dollar of government funding – respondents were less likely to select profiles that obtained a higher share of their funding from out-of-pocket contributions. The final specification for the population-average, 'lives saved' model correctly classified 76% (955/1257) of unweighted choices in favour of profile A (NOT profile B) and 78% (836/1072) of unweighted choices in favour of profile B.

Parameter estimates from the 'life-years saved' model are broadly consistent with those from the 'lives saved' model, with differences in the magnitude and sign of coefficients on AgeGrp dummies being attributable to the fact that duration of effect is now being captured by our measure of incremental effectiveness. Specifically, estimated regression coefficients on the AgeGrp dummies suggest a weaker preference for interventions targeting young children and young adults than was suggested by the 'lives saved' model. The final specification for the population-average LYs saved model correctly classified 76% (958/1257) of unweighted choices in favour of profile A (NOT profile B) and 77% (830/1072) of unweighted choices in favour of profile B.

### Estimating willingness to trade and willingness to pay

Table 6 summarises marginal effects for lives saved population-average model. Marginal effects were calculated at the median for each attribute and reflect a discrete change between categories for dichotomous and categorical variables. Willingness to pay (WTP) is derived as described above by taking the ratio of marginal effects. Using this approach, WTP for an additional life saved is estimated at: (0.0084590/0.0015023)*100,000 = AUD563,070 where the marginal effect on the cost attribute is expressed in multiples of AUD100,000. Note that this estimate is almost identical to the ratio of the parameter estimates: (0.00338446/0.0060109)* 100,000 = AUD563,054. For the main effects model estimated here, minor differences between WTP for a life saved by the median program and any other program arise simply as a function of the dependence between marginal effects and the value of covariates for the logistic regression model.

**Table 6:** Marginal effects for population average models

Predictor	∂ U_B _- U_A_/∂ x_j_	SE	95%CI	x_j_	∂ U_B _- U_A_/∂ x_j_	SE	95%CI	x_j_
	Lives saved				Life-years saved			

Cure(B – A)^~^	-0.2118	0.028	(-0.27,-0.16)	0	-0.2082	0.026	(-0.26,-0.16)	0
AgeGrp1(B – A)^†^	0.3222	0.037	(0.25, 0.39)	0	0.1862	0.036	(0.12, 0.26)	0
AgeGrp2(B – A)^†^	0.1484	0.034	(0.08, 0.22)	0	0.0750	0.033	(0.01, 0.14)	0
AgeGrp4(B – A)^†^	-0.0952	0.028	(-0.15,-0.04)	0	0.0047	0.032	(-0.06,0.07)	0
Evidence(B – A)^~^	0.1714	0.023	(0.13, 0.22)	0	0.1643	0.023	(0.12, 0.21)	0
Fault(B – A)^~^	-0.1455	0.024	(-0.19,-0.10)	0	-0.1640	0.026	(-0.21,-0.11)	0
$Private(B – A)^	-0.0014	0.001	(-0.00,-0.00)	0	-0.0019	0.001	(-0.00,-0.00)	0
Effect(B – A)^‡^	0.0085	0.001	(0.01, 0.01)	0	0.0002	0.000	(0.00, 0.00)	0
$Cost(B – A)^	-0.0015	0.000	(-0.00,-0.00)	0	-0.0014	0.000	(-0.00,-0.00)	0
HlthCard*Q^~^	-0.0114	0.005	(-0.02,-0.00)	0	-0.0113	0.005	(-0.02,-0.00)	0
(SIEFA_Econ*Q)/1000	0.0173	0.006	(0.01, 0.03)	5.4	0.0198	0.006	(0.01, 0.03)	5.4

^Dollar values expressed in AUD100,000s.

^†^Reference category is 'working-age adults'. First, second and fourth dummies denote 'young children', 'young adults' and 'older-age retirees', respectively. Here, ∂ U_B _- U_A_/∂ x_j _is for discrete change from reference category to age-group denoted by relevant dummy variable.

^‡^Effect(B – A) gives the incremental effectiveness of profile B compared to profile A defined in terms of terms of lives saved for the 'lives-saved' model and life-years saved for the 'life-years saved' model.

^~ ^For dichotomous variables, ∂ U_B _- U_A_/∂ x_j _is for discrete change in dummy variable from 0 to 1.

Willingness to pay for a life saved by different types of program should be distinguished from WTP for switching between different types of intervention. The willingness to trade or marginal rate of substitution between any two profiles can be derived as the ratio of their marginal effects on the latent dependent variable. Willingness to pay for switching from a preventative intervention targeting young children to a curative intervention targeting older-age retirees, for example, can be derived by calculating the difference in the predicted value of the latent dependent variable when values of Cure(B – A), AgeGrp1(B – A) and AgeGrp4(B – A) are modified, before dividing through by the marginal effect on incremental cost. Because marginal effects are a function of the value taken by other covariates, the difference in the predicted value of the dependent variable for changes across more than one attribute will only be approximated by an addition over individual marginal effects. Using this approach, WTP for a preventative intervention in young children that saves the same number of lives (median = 30 lives for both profiles) as a curative intervention in the elderly is estimated at (0.5573317/0.0015023)*100,000 = AUD37.1million. Because respondents are selecting between programs, the scale of the programs included in the choice scenarios will influence WTP values.

While it is not possible to report WTP values for all possible programs for a universe of 4096 programs (2*2*2*4*2*4*4*4), a WTP for substitution between any two profiles can be easily recovered from the results summarised in Tables 4 and 5. First, substitute appropriate values for each level of each attribute into the regression equation given in Table 5 to obtain log-odds for each profile. Second, recover the predicted probabilities for each profile as e^log-odds^/(e^log-odds ^+ 1) and take the difference in predicted probabilities between the two profiles. Finally, divide the difference in predicted probabilities through by the marginal effect on incremental cost calculated for the median program from Table 6 (or for the baseline program if different from the median program).

Table 6 also reports marginal effects for the LYs saved version of the population-average model, with incremental effectiveness expressed in terms of LYs saved to permit willingness to pay to be calculated for LYs as well as lives. Taking the ratio of marginal effects on incremental effectiveness and incremental cost, WTP for an additional LY saved is estimated at: (0.0001570/0.0014147)*100,000 = AUD11,098. Willingness to pay for saving the life of a 5 year old with a life-expectancy averaging a further 76.3 years in the Australian population [38,39] is then estimated at AUD838,567. Willingness to pay for saving the life of a 18 year old with a life-expectancy averaging a further 63.5 years in the Australian population [38,39] is estimated at AUD702,223. Willingness to pay for saving the life of a 40 year old with a life-expectancy averaging a further 42.3 years in the Australian population [38,39] is estimated at AUD469,443. Willingness to pay for saving the life of a 70 year old with a life-expectancy averaging a further 15.7 years in the Australian population [38,39] is calculated at AUD174,255. These figures differ slightly from those that would be obtained by multiplying the value of a life-year saved by the remaining life-expectancy because the marginal effects on incremental effectiveness and incremental cost are calculated for a program targeting the appropriate age group rather than for the median program.

## Discussion & Conclusion

The marginal effects and marginal rates of substitution reported here confirm the relevance of non-health arguments when individuals prioritise over health states. Specifically, a number of non-health attributes were individually significant in determining stated-preferences including the life-stage or age-group of the target population, whether the intervention was curative or preventative, the strength of evidence regarding risks and benefits attributed to the intervention, and the extent to which beneficiaries have contributed to their illness via voluntary adoption of risky lifestyle. The explanations given in support of stated-preferences were broadly consistent with these findings and suggested that respondents were making principled decisions based on due consideration of the alternatives presented to them.

For the main effects model estimated here, the effect of each attribute is assumed orthogonal to the effect of all other attributes with no quantitatively important interactions between attributes. While we were restricted to estimating main effects, it is possible that quantitatively important interactions may exist between health and one or more of the non-health attributes. Specifically, it might be the case that some of the marginal effect of incremental effectiveness on the latent dependent variable has been picked up in the coefficients on the age group and cure/prevention dummies. All else being equal, we would expect interventions targeting young children to save more LYs per life saved than interventions targeting the elderly. Likewise, respondents may have valued curative interventions more highly than preventative interventions because they thought the threat to life more immediate in the case of a curative intervention (implying that a curative intervention would save more discounted LYs per life saved than a preventative intervention). Any interactions along the lines described above are not separately identifiable from the main effects using the main effects-only design employed here.

While marginal effects for age/life-stage dummies in the lives saved model may be partly attributable to capacity to benefit, marginal effects from the LYs saved model were consistent with a preference for interventions targeting young children and young adults even after correcting for duration of benefit. Marginal effects also suggested a weaker preference for interventions targeting young children and young adults in the life-years saved model than in the lives saved model. Note that this is exactly what we would expect to happen after controlling for the higher weight attached to saving the lives of those with a longer life-expectancy. After expressing incremental effectiveness in terms of life-years rather than lives saved, the higher weight attached to saving the lives of those with a longer life-expectancy is picked up by the Effect(B – A) variable and the marginal effect on Effect(B – A) must be multiplied by life-expectancy when calculating willingness to pay. Marginal effects from the life-years saved model are broadly consistent with age-based distributive preferences reported elsewhere [3,4,41] but give greater weight to the lives of children than the life-cycle model of net productive contribution to society that underpins the DALY (disability-adjusted life-year) age-weights [9].

Likewise, while it is possible that the cure/prevention distinction and strength of evidence distinction were interpreted by respondents as proxies for the magnitude of health gain, these variables remained significant after correcting for duration of benefit. Finally, interactions between health and non-health attributes are plausible for some but not all non-health attributes. Note, in particular, that preferences over health programs were also dependent upon the extent to which beneficiaries contributed to their illness via voluntary adoption of risky lifestyle. Olsen et al [42] suggest a number of ethical bases that might justify a higher or lower priority based on fault including desert and merit or personal responsibility but do not link the notion of fault to potential health gain. Our findings therefore confirm that a trade-off exists between cost, effectiveness and non-health arguments, despite the potential for uncontrolled interactions between health and non-health arguments.

That said, it is true that the presence of any uncontrolled interactions between health and non-health attributes in the present study may have biased parameter estimates. Note in particular that the WTP estimates reported above for the value of a life and LY saved are at the lower limit of published estimates [12,13] and that some of the marginal effect of incremental effectiveness may have been picked up by the age group and cure/prevention dummies. While we have attempted to correct for duration, it is worth noting that the LYs saved model makes various assumptions in order to express incremental effectiveness in terms of LYs saved. Specifically, an estimate of LYs saved was obtained by combining estimates of population by age and sex [38] with life-expectancies at each life-stage for the Australian population [39]. This calculation required an exact age to be specified for each life-stage as follows: 'young children': 5 yrs, 'young adults': 18 yrs, 'working-age adults': 40 yrs, 'older-age retirees': 70 yrs. While results from the lives saved and LYs saved models are broadly consistent, it might be the case that respondents' based their valuations on life-expectancies that differed from ABS life-tables [39] or that respondents assumed a higher or lower exact age than we did to characterise each life-stage such that our estimate for the marginal effect on incremental effectiveness might remain an underestimate even after correcting for duration.

In this context, it is worth considering the available evidence regarding correspondence between subjective and objective evaluations of life-expectancy. Hurd and McGarry [43] found that respondents to the US Health and Retirement Study (HRS) who were aged 51 to 61 years at the time of interview (n = 7946) provided subjective evaluations of probability of survival to ages 75 and 85 that, when averaged across all respondents, correlated very closely to life-tables and that co-varied with socio-economic status, health status and risk factors in a manner consistent with objective data. Note, however, that "two measures can be perfectly correlated but have poor agreement" [[44] p977] and closer inspection of the available evidence suggests that relatively stable biases might be embedded in subjective evaluations. Data from the Hurd and McGarry [43] study, for example, suggest that men might have a tendency to overestimate their life-expectancy whereas women tend to underestimate their life-expectancy. Consistent with these findings, Mirowsky [45] identified several points of divergence between subjective and actuarial estimates of life-expectancy in a sample of 2037 Americans aged 18–95. Specifically, males typically evaluated their life-expectancy at approximately 3 years longer than was predicted by life-tables and blacks over-estimated their life-expectancy by approximately 6 years. It is, however, unlikely that the consistent biases identified in the literature are relevant in interpreting results reported here because no such consistent bias has been identified by age or life-stage. For example, Mirowsky [45] found that "differences across age groups in mean subjective longevity and life expectancy track the corresponding actuarial estimates well" (p975) and note that "subjective estimates overall show an optimistic bias of about one year that does not increase or decrease with age" (p976).

Our study is also subject to limitations that might limit the applicability of findings. First, recall that our data reflect the preferences of a relatively wealthy, well-educated segment of the Australian population employed in relatively high-skilled occupations. Policy-makers seeking to apply lessons learnt from the present study should consider carefully the similarities and differences between our study sample and their target population. Second, our study considered only life-saving programs and excluded a number of potentially relevant attributes in an attempt to address comments from the pilot surveys regarding the difficulty of making tradeoffs over even a relatively small number of attributes and in recognition of the potential for cognitive overload when individuals are faced with abstract and complex decisions [29-31]. Comments on a number of surveys also suggested that some respondents may have had difficulties interpreting the $Private(B – A) attribute describing the share of patient contributions to the total cost of the program. Specifically, some respondents may have interpreted the private share to have been additional to the cost of the program reflected in the $COST(B – A) attribute.

Finally, the two-alternative, forced choice format of the discrete choice scenarios presented to respondents does not correspond to the typical resource allocation problem facing decision makers where resources might be allocated across more than two options and where decision-makers typically retain the right to reject/accept all submissions for funding. We settled on the two-alternative forced choice format because our piloting suggested that the two alternative forced choice was difficult enough without introducing additional options, because a no-choice option may have proved too attractive to respondents when faced with difficult tradeoffs, and because recent findings suggest that parameter estimates from forced choice formats should be unbiased despite the fact that stated-preferences reflect a simplified view of real-world decision-making [46].

Despite these limitations, our findings provide a unique insight into the tradeoffs that individuals make when prioritising health programs. The marginal effects reported above and the implied marginal rates of substitution between incremental cost, incremental effectiveness and various non-health arguments confirm that community values are inconsistent with simple health maximisation. That said, it is true that respondents were more likely to select less costly, more effective interventions – confirming that it is an adjustment to, rather than an outright rejection of, simple health maximisation that is required. Nord [19] coined the term cost-value analysis to describe one possible means of making such an adjustment wherein QALYs are replaced with value-weighted QALYs. Priority setting then becomes an exercise in 'value' maximisation rather than simple QALY maximisation.

To date, attempts to modify funding thresholds or value-weight outcomes [19] have typically adjusted for only a narrow subset of potentially relevant non-health characteristics such as distribution [20], age [9] or severity [21] with age-, severity- or equity-weights typically derived in isolation of other potentially relevant non-health characteristics. The few studies that have quantified tradeoffs across a set of attributes that includes *multiple *non-health characteristics relate to resource-poor settings and reflect the preferences of policy- and decision-makers rather than directly accessing community preferences. For example, Baltussen et al [47,48] conducted a discrete choice experiment in 30 persons involved in policy- and decision-making in Ghana's health sector to obtain stated-preferences over programs defined by 'cost-effectiveness', 'poverty reduction', 'severity of disease', 'age of target group', 'budget impact' and 'individual health effect'. Respondents in the Baltussen et al [47,48] study were more likely to select cost-effective programs for severe diseases that reduce poverty and target younger age-groups. Similarly, Baltussen et al [49] conducted a discrete choice experiment in 66 policy-makers and health professionals involved in mid-level health care management and public health provision in Nepal's health sector to obtain stated-preferences over programs defined by 'cost-effectiveness', 'poverty reduction', 'severity of disease', 'age of target group', 'number of potential beneficiaries' and 'individual health effect'. Respondents in the Baltussen et al [49] study were more likely to select cost-effective programs for severe diseases that offer large individual health benefits to many beneficiaries, reduce poverty and target the middle-aged.

These recent attempts to derive a more comprehensive set of tradeoffs over health and non-health attributes constitute an advance on age-, severity- or equity-weights derived in isolation. Specifically, the approach taken in the present study and in recent work by Baltussen et al [47,48] offers some promise in obtaining a set of weights that would avoid the double-counting that might arise when weights are developed in a piecemeal fashion and then applied one upon the other [22]. While it is difficult to draw comparisons across settings given the socio-cultural determinants of community preferences and the extent of between context variation in GDP per capita, comparison between our findings and those reported by Baltussen et al [47,48] suggests that non-health attributes may have a role to play in priority setting *irrespective of context*. The task now is to build on the lessons learnt, employing larger fractional or full factorial designs to explicitly account for all potentially relevant main effects and interactions between health and non-health attributes. It is, however, worth emphasising that, while there is no consensus in the literature regarding the tradeoff between complexity and completeness in the conduct of discrete choice experiments [29], our piloting and feedback from the survey sample suggests that many respondents would have difficulty with the complex and abstract scenarios that would be required to derive a comprehensive set of weights that accounts for all relevant main effects and interactions.

Setting aside questions with regards feasibility and acceptability, there is the prior matter of whether the costly and complex exercise of deriving a universal set of value-weights is the most efficient use of research dollars. One possible alternative is to eschew attempts to derive a value-weighted QALY that could be universally applied and, to instead, directly value the benefits derived from each evaluated intervention in dollar-terms. Note that constraints with regards cognitive demands are less likely to bind where stated-preferences are sought over a limited set of relatively homogeneous real-world alternatives than when comparisons are drawn across the entire choice set. Likewise, descriptions of programs and program attributes can be made much less abstract when comparing specific alternatives in dollar-terms. While the use of cost-benefit analysis for the evaluation of health care interventions requires careful negotiation of relatively well-known pitfalls [50-52], the difficulties of directly valuing health benefits in dollar-terms should be compared – not against the simplified partial approach to valuing outcomes that is embedded in cost-utility analysis – but against the difficulties of obtaining a comprehensive set of weights for use in cost-value analysis.

## List of abbreviations used

95%CI: 95% confidence interval; ABS: Australian Bureau of Statistics; AUD: Australian Dollar; cov: covariance; DALY: disability-adjusted life year; HlthCard: health card; ICC: intra-cluster correlation coefficient; LY: life-year; QALY: quality-adjusted life year; SD: standard deviation; SEIFA: socio-economic indices for areas; WTP: willingness-to-pay; YRS: years.

## Competing interests

The authors declare that they have no competing interests.

## Authors' contributions

DM participated in the design of the study, coordinated the data collection, completed the data analysis, and interpretation of results, and drafted the manuscript. LS participated in the design of the study and suggested edits and revisions to the manuscript. Both authors read and approved the final manuscript.
